# Prevalence and Factors Associated with Road Traffic Crash among Taxi Drivers in Mekelle Town, Northern Ethiopia, 2014: A Cross Sectional Study

**DOI:** 10.1371/journal.pone.0118675

**Published:** 2015-03-17

**Authors:** Nigus Gebremedhin Asefa, Lalit Ingale, Ashenafi Shumey, Hannah Yang

**Affiliations:** 1 Dr. Tewelde Legesse Health Sciences College, Mekelle, Tigray, Ethiopia; 2 Mekelle University, College of Health Sciences, Mekelle, Tigray, Ethiopia; 3 Department of International Health, Johns Hopkins Bloomberg School of Public Health, Baltimore, Maryland, United States of America; National Institute of Health, ITALY

## Abstract

**Objectives:**

The 2013 World Health Organization Status Report on Road Safety estimated that approximately 1.24 million deaths occur annually due to road traffic crashes with most of the burden falling on low- and middle-income countries. The objective of this research is to study the prevalence of road traffic crashes in Mekelle, Tigray, Northern Ethiopia and to identify risk factors with the ultimate goal of informing prevention activities and policies.

**Methods:**

This study used a cross-sectional design to measure the prevalence and factors associated with road traffic crashes among 4-wheeled minibus (n = 130) and 3-wheeled Bajaj (n = 582) taxi drivers in Mekelle, Ethiopia. Bivariate and multivariate logistic regression were used to evaluate the association between risk factors and drivers’ involvement in a road traffic crash within the 3 years prior to the survey.

**Findings:**

Among the 712 taxi drivers, 26.4% (n = 188) of them reported involvement in a road traffic crash within the past 3 years. Drivers who listened to mass media had decreased likelihood of road traffic crash involvement (AOR = 0.51, 0.33–0.78), while speedy driving (AOR = 4.57, 3.05–7.44), receipt of a prior traffic punishment (AOR = 4.57, 2.67–7.85), and driving a mechanically faulty taxi (AOR = 4.91, 2.81–8.61) were strongly associated with road traffic crash involvement. Receiving mobile phone calls while driving (AOR = 1.91, 1.24–2.92) and history of alcohol use (AOR = 1.51, 1.00–2.28) were also associated with higher odds of road traffic crash involvement.

**Conclusion:**

The results of this study show that taxi drivers in Mekelle habitually place themselves at increased risk of road traffic crashes by violating traffic laws, especially related to speedy driving, mobile phone use, and taxi maintenance. This research can be used to support re-evaluation of the type, severity, and enforcement of traffic violation penalties.

## Introduction

Road traffic crash (RTC) is defined as a collision between two or more vehicles, between vehicles and pedestrians, between vehicles and animals, or between vehicles and fixed obstacles[1]. Low- and middle-income countries (LMICs) in particular experience a disproportionate burden of RTC-related injuries bearing over 90% of the world’s traffic related-fatalities even though they have less than 50% of the world’s registered vehicles[2]. As vehicle ownership in LMICs continues to rise, the public health problem of RTCs will only gain momentum. It is estimated that RTCs cost LMICs more than $100 billion USD every year, or 1–2% of their gross national product (GNP) due to premature death, disability, medical expenses, loss of productivity, and material damages [3]. Despite these major impacts upon global health and economic development, RTCs and related injuries tend to be under-prioritized in LMICs [2].

At 24.1 deaths per 100,000 populations in 2010, the continent of Africa has the highest traffic-related mortality rate [3]. Among African countries, Ethiopia has a relatively high burden [4]. The UN Economic Commission for Africa reported that 15,086 road traffic crashes occurred in Ethiopia in 2008 resulting in a mortality rate of 95 deaths per 10,000 vehicles, and causing losses of over 82 million Ethiopian Birr ($7.3 million USD) [4].

It is challenging to accurately estimate the public health burden and causes of RTCs in Ethiopia for a number of reasons. Most reported statistics are based on police data, which is believed to underreport true rates of death from RTCs in many countries [3, 5–7]. In addition, most academic studies conducted in Ethiopia are restricted to Addis Ababa or are part of a large multi-country study. For example, studies have shown that speedy driving accounted for 13–50% of RTCs in Ethiopia, Ghana, and Kenya [4, 8]. Risk factors such as poor vehicle maintenance (including tires, brakes, and lights), choked roads, and driving old vehicles were identified in Ethiopia, India, and Libya [9–11].However, it should be noted that the causes of road crashes are normally multi-factors.

A twenty-year report from the Tigray Police Commission and Tigray Construction, Road, and Transport Bureau documented a significant increase in the number of RTCs occurring annually in Tigray region, from 336 crashes in 1993 to 1,304 in 2007. The report stated that 87% of the crashes were related to drivers’ risky behavior, 5% were due to vehicle problems, 3% due to a pedestrian problem, and the remaining 5% were due to poor road and environmental conditions[12].

According to the same report, taxis constitute 16.55% of the total vehicles in the region [12]. However, no academic studies have focused on taxi drivers despite the fact that taxis are the primary mode of public transportation within all major Ethiopian cities. Therefore, the purpose of this research is to fill the information gap on RTCs in Ethiopia by studying the prevalence and risk factors associated with RTC involvement among taxi drivers in Mekelle Town, the capital city of Tigray National Regional State in northern Ethiopia.

## Methods

This study used a cross-sectional study design. The sample size was determined using two-population proportion formula with prevalence estimates taken from a study conducted in Vietnam [13]. Assuming 95% two-sided confidence level and a power of 80%, the total sample size was calculated as 761, including 10% non-response rate. During the study period, there were 728 four-wheeled minibus and 3,455 three-wheeled Bajaj taxis in Mekelle [12]. A sampling frame of all taxis was obtained from Tigray Construction, Road, and Transport Bureau and participants were proportionally selected using systematic random sampling.

Data was collected using a semi-structured questionnaire prepared in English and then translated to the local language, Tigrigna. It was piloted on 5% (n = 30) taxi drivers to check the consistency and understandability of the question and slight modifications in the data collection process were made based on frequent data quality checks. The final version of the questionnaire is included in S1 Appendix.

The dependent variable in this study was self-reported history of involvement in one or more RTC(s) within the past three years. The independent variables were several factors related to the driver and the condition of their taxi vehicle.

The data were entered into EPI Info version 3.5 and analyzed using SPSS version 20 statistical package. Bivariate logistic regression analysis was carried out to distinguish the independent effect of each variable. Using multivariate logistic regression analysis, 95% CI and adjusted odds ratios (AORs) were computed in order to identify any statistically significant associations between risk factors and RTC involvement. The level of statistical significance was set at P<0.05.

### Ethics statement

Ethical clearance was obtained from the Institutional Review Board of Mekelle University, College of Health Sciences. This study was approved by the Institutional Review Board of Mekelle University, College of Health Sciences.Written concent was obtained from all the participants and they were informed that participation is surely voluntary and they can withdraw any time during conducting the study.

## Results

### Taxi driver characteristics

A total of 712 taxi drivers participated in the study, resulting in a response rate of 93.6%. The median age of taxi drivers was 26.0 years with respondents ranging from 19 to 69 years old. As seen in Table 1, most of the drivers were between the ages of 25–44 years (n = 387, 54.4%), singles (n = 478, 67.1%), and Orthodox Christians (n = 646, 91.0%). The median monthly income of taxi drivers was 2000.00 EthB and nearly half of the drivers were self-employed (n = 400, 56.2%) meaning that they own their own taxi vehicle.

**Table 1:** Socio-economic characteristics of taxi drivers.

Variables	Frequency (n)	Percent (%)
**Age category (years)**		
< 25	301	42.3
25–44	387	54.4
> 44	24	3.4
**Marital status**		
Single	478	67.1
Married	225	31.6
Divorced	9	1.3
**Religion**		
Orthodox	646	90.7
Muslim	59	8.3
Other	7	1.0
**Level of education**		
Can read and write	17	2.4
1^st^- 8^th^ Grade complete	139	19.5
9^th^-12^th^ Grade complete	409	57.4
Diploma and above	147	20.7
**Listens to mass media**		
Yes	482	67.7
No	230	32.3
**Monthly Salary (Ethiopian Birr, EthB)**		
< 1000	129	18.1
1000–2000	268	37.6
2001–3000	240	33.7
> 3000	75	10.5
**Owns their own residence**		
Yes	95	13.3
No	617	86.7
**Owner of the taxi**		
Self	400	56.2
Employer	312	43.8

More than 75% of the drivers had the minimum required driving license (1^st^ level) to drive a taxi, while only 2% of drivers had reached 4^th^ to 5^th^level. Only 16.4% of drivers reported using a seat belt as required by law, but it should be noted that there is no built-in seat belt in Bajaj taxis. About half of the taxi drivers, (n = 237, 50.1%) reported that they had received 1–2 punishments for violating traffic laws in the past. Over 20% of the drivers reported receiving 3–4 punishments (n = 97, 20.5%), and nearly one third of the drivers had been punished more than 4 times (n = 139, 29.4%). Over half (n = 357, 50.1%) of the taxi drivers reported that they drove above the national urban speed limit of 60 km/hr for different reasons. About 22% of taxi drivers reported that they received mobile calls while driving, rather than stopping the vehicle to receive the call. Approximately one-third of the drivers reported a history of ever using alcohol (n = 250, 35.1%). Out of these,73%reported having operated their taxi within 3 hours of drinking alcohol. Only 9% of the drivers reported a habit of chewing the stimulant plant *Khat* while driving.

### Vehicle characteristics

The majority of the study participants (n = 582, 81.7%) drove Three-Wheeled Bajaj taxis, while the remainder drove four-wheeled minibus taxis. The median age of Three-Wheeled taxis was 3 years (IQR, 1.87–5). Similarly, the median age of Four- Wheeled taxis was 7 years (IQR, 4–11). Two-thirds of the drivers (n = 498, 69.9%) reported encountering mechanical problems with their taxis including malfunctioning breaks (n = 126, 17.7%), steering system failure (n = 76, 10.7%), lighting system problems (n = 194, 27.2%), and poor quality tires (n = 346, 48.6%).

### Factors related to RTC involvement

Among a total of 712 taxi drivers, 188 self-reported involvement in an RTC in the past three years (2011–2013) for a prevalence of 26.4%. Study participants reported 10 human deaths, 13 animal deaths, 107 non-fatal injuries, and 112 property damage. Of the drivers who had experienced RTC, 13.8% mentioned rainy weather conditions as a contributing cause.

After applying both bivariate and multivariate logistic regression, six variables were found to be independent predictors of road traffic crash involvement. Listening to mass media (like TV and FM programs) was found to be a protective factor, while a history of traffic punishment, speedy driving, receiving mobile calls without stopping the vehicle, history of alcohol use, and driving a taxi with mechanical problems were found to increase the odds of RTC involvement (Table 2). Driving a taxi with mechanical problems showed the strongest association with the outcome (AOR = 4.91, 95% CI: 2.81–8.61), followed by speedy driving (AOR = 4.76, 95%CI: 3.05–7.44), and having a history of traffic punishment (AOR = 4.58, 95%CI: 2.67–7.85) (Table 2).

**Table 2:** Factors related to RTC among taxi drivers.

	In the last 3 years, have you been involved in a road traffic crash?				
Variables	Yes (%) (N = 188)	No(%) (N = 524)	COR (95% CI)	AOR (95%CI)	p-value
**Listens to mass media**					
No	91(39.57)	139(60.43)	1	1	
Yes	97(20.12)	385(79.88)	0.38(0.27–0.54)	0.51(0.33–0.78)	0.002
**Salary (ETB)/month**					
1000–2000	57(21.27)	211(78.73)	1	1	0.944
Less than 1000	26(20.16)	103(79.84)	0.93(0.56–1.57)	1.04(0.56–1.93)	0.902
2001–3000	77(32.08)	163(67.92)	1.75(1.17–2.61)	0.99(0.61–1.62)	0.970
3001–4000	21(38.89)	33(61.11)	2.36(1.27–4.38)	1.31(0.62–2.75)	0.477
Above 4000	7(33.33)	14(66.67)	1.85(0.71–4.80)	1.29(0.40–4.10)	0.666
**First aid trained**					
No	128(28.96)	314(71.04)	1	1	
Yes	60(22.22)	210(77.78)	0.70(0.49–0.99)	0.91(0.60–1.40)	0.645
**Punished for disregarding of traffic rules**					
No	20(8.37)	219(91.63)	1	1	
Yes	168(35.52)	305(64.48)	6.03(3.68–9.89)	4.58(2.67–7.85)	<0.001
**Drives above the speed limit**					
No	36(10.14)	319(89.86)	1	1	
Yes	152(42.58)	205(57.42)	6.57(4.39–9.83)	4.76(3.05–7.44)	<0.001
**Method of receiving mobile calls**					
Stopping	100(21.41)	367(78.78)	1	1	
Accept or reject without stopping	88(35.92)	157(64.08)	2.06(1.46–2.90)	1.91(1.24–2.92)	0.003
**History of alcohol use**					
No	102(22.08)	360(77.92)	1	1	
Yes	86(34.40)	164(65.60)	1.85(1.32–2.60)	1.51(1.002–2.28)	0.049
**Chewing*Khat***					
No	155(23.85)	495(76.15)	1	1	
Yes	33(53.23)	29(46.77)	3.63(2.14–6.18)	1.88(0.99–3.60)	0.053
**Drives a taxi with mechanical problems**					
No	19(2.67)	195(27.39)	1	1	
Yes	169(33.94)	329(66.06)	5.27(3.18–8.75)	4.91(2.81–8.61)	<0.001

## Discussion

The study focused specifically on 3-and 4-wheeled municipal taxi drivers, revealing that around one quarter (26.4%) of them had been involved in an RTC in the past three years. This is similar to the prevalence reported among taxi drivers in both Vietnam and South Africa [13,14]. The study results suggest that taxi drivers in Mekelle can be effectively reached through mass media campaigns, but there are several other factors disposing drivers to a higher risk of RTC. Results from this study can be used to inform the design of preventative interventions, but further research must be conducted on the economic, regulatory, and socio-cultural issues that affect RTC risk exposure.

Although Ethiopia has passed regulations at national level requiring seat belts in all new and imported cars [3], this study showed that poor taxi maintenance remains a persistent challenge, consistent with research findings in other LMICs [15,16]. As Ethiopia moves forward with measures to ensure safer vehicles, regulations should account for the fact that taxis (including 3-wheeled Bajaj) comprise a large proportion of the country’s transportation sector ensuring that the legislation is fully inclusive. The country has also passed national laws prohibiting mobile phone (hand-held and hands-free) use while driving [3], however, 35% of the study participants reported using their mobile phones while driving. Since mobile phone use is consistently a risk factor for RTC [17,18], the enforcement of these laws should be re-evaluated and safe driving practices should be promoted.

This study identified an association between speedy driving and RTC, but there is mixed findings about this topic in the existing literature. Similar to this study, speedy driving was associated with RTC in China [19], Oman [16], Iran [20] and Nepal [21]. However, it was not associated with RTC in Vietnam [13] and Australia [22].Economic status of the taxi drivers, road user mix, traffic pattern or density, legal framework, and other socio-cultural issues may affect whether speedy driving is a perceived or actual cause of road traffic crash in various countries and contexts.

Previous studies in the USA have indicated that alcohol and other CNS stimulants are highly related with RTC [23,24], consistent with the findings of this study. However, it is important to clarify the data definition of “alcohol use” in this study as it may affect the interpretation of this finding. This study included all drivers who reported any lifetime history of using alcohol as “alcohol users”, rather than making a distinction for drivers who reported actually driving under the influence of alcohol. In other words, the definition of “alcohol use” used in this study was not directly linked with the operation of the taxi. This may have weakened the association between the variable alcohol use and the study outcome in the analysis.

Nearly 90% of drivers reporting RTC involvement in this study had a history of at least one traffic punishment, and over 50% of the total study participants had received a punishment as well. This reveals a high level of risky driving among the study population, and a persistent habit of violating traffic laws. Therefore, the results of this study may have implications for modifying the type, severity, and enforcement level of traffic violation punishments used in the Ethiopian context.

Limitations of this study include selection bias, recall bias, and survival bias. Taxi drivers are a highly mobile population, which can create inconsistency in the selection of study participants using stratified systematic random sampling. The study was based on self-report of the past three years and therefore may have been subject to recall bias. In addition, the study only included living drivers, which through survival bias can lead to under-estimation of the effect magnitude.

## Conclusion

Several of the risk factors for road traffic crash that were identified in this study relate to direct and habitual violation of existing traffic laws, including vehicle maintenance standards, speedy driving, drink-driving, and mobile phone use while driving. The results of this study suggest the need for a re-evaluation of the appropriateness and implementation status of the existing legal framework. Alternative forms of punishment could be considered as well as increased policing and enforcement, but the system must be supported by continual monitoringand investment of sufficient financial and human resources.

## Supporting Information

S1 Appendix Study Questionnaire in English and Tigrigna. (PDF)

S1 Dataset Questionnaire ResultsData in SPSS Format. (SAV)
