# Supplementary material for: Prevalence and Factors Associated with Road Traffic Crash among Taxi Drivers in Mekelle Town, Northern Ethiopia, 2014: A Cross Sectional Study
Source: PLoS One. 2015 Mar 17;10(3):e0118675. doi: 10.1371/journal.pone.0118675 (PMC4363695; doi:10.1371/journal.pone.0118675)
Supplement: S1 Appendix — (PDF) [file pone.0118675.s001.pdf]

Questionnaire_S1.File

Part-One. Socio-demographic information of drivers

101. What is your age? ______Years

102. What is your religion?

1. Orthodox 3. Muslim
2. Catholic 4. Protestant 5. Other, ______________

103. What is your marital status?

1. Single 3. Divorced
2. Married

104. How many children do you have?

1. 0 children
2. 1-2 children 2. Above 2 children

105. What is your level of education?

1. Read and write 3. Secondary school
2. Elementary school 4. Diploma and above

106. How many family members do you support?

1. 0
2. 1-2
3. 3-4
4. 5 and above.

107. What is your monthly salary while driving? ______Birr

108. Do you have your own residence?

1. Yes 2. No

Part-Two. Driver's characteristics

201. Have you got first aid training? 1. Yes 2. No

202. Do you have life insurance? 1. Yes 2. No

203. Do you use your seatbelt? 1. Yes 2. No

204. If your answer to question number 203 is YES, how do you use your seat belt?

1. Always 3. Sometimes
2. Most of the time

205. If your answer to question number 203 is NO, what is your reason?

1. I fed up
2. It is not functional
3. It does not have benefits
4. No seat belt at all
5. Other, specify, ____________________

206. Do you drink any alcoholic beverages? 1. Yes 2. No

207. If your answer to question number 206 is "**Yes**", how often do you drive within 3 hours of having one or more alcoholic drinks?

1. Very often 3. Seldom
2. Occasionally 4. Never

208. Have you ever been punished by traffic police for any disregarding traffic rules?

1. Yes 2. No.

209. If your answer to question number 208 is "**Yes",** how many times?

1. One-Two 2. Three-four 3. Above 4

210. Do you chew *Khat*? 1. Yes 2. No

211. If your answer to question number 210 is "**Yes",** how often do you chew *Khat* while driving?

1. Always 3. Sometimes
2. Most of the time

212. If your answer to question number 210 is **"Yes",** what do you think is the benefit of chewing *Khat* while driving?

1. It energizes me
2. For pleasure
3. I feel free
4. Other, specify ________________

213. Do you drive more than the limited speed? 1. Yes 2. No

214. If your answer to question number 213 is **"Yes",** how often do you drive more than the limited speed?

1. Very often 3. Occasionally
2. Seldom

215. If your answer to question number 213 is **"Yes",** what do you think is the reason?

1. For short queue
2. To increase income
3. Racing with another driver
4. Other, specify ______________________

216. If your cell phone rang while driving, how you receive your calls?

1. Reduces speed and receive calls 3. Receiving calls at normal speed
2. Stop driving and receive calls 4. I disconnect ringing

217. Until what time do you drive at night? ________ pm

218. Do you listen FM and TV programs?

1.Yes 2. No

219. What is your level of driving license?__________

220. What is your driving experience as a taxi driver? _______________ Years

221. Where did you train driving license?____________________________________

Part-Three. Vehicle's condition

301. How many years are the services of your vehicle? __________Years

302. What type of vehicle you drive currently?

1. Three-wheeled taxi2. Four wheeled taxi

303. Who is the owner of this vehicle?

1. Myself 2. My employer

304. Does your taxi/Baja encounter a mechanical problem? 1. Yes 2. No

305. If your answer to question number 304 is "**Yes",** what common mechanical problem does the vehicle encounter?

1. Brake 4. Lighting
2. Steer 3. Tire

Part-Four. About road traffic accident condition

401. Have you ever had road traffic accident in the last 3 years?

1. Yes 2. No

1. If your answer to question number 401 is **"Yes",** what was the cause of the accident? More than one choice is possible.Over-speed driving
2. Alcohol driving
3. Khat chewing
4. Pedestrians carelessness
5. Failure to follow the right hand rule
6. Failure to give way for pedestrian
7. Phone use while driving
8. Following too close
9. Quality of road
10. Vehicle's mechanical problem
11. Other, specify ______________

402. If your answer to question number 401 is **"Yes",** what type of collision was it?

1. With human/pedestrian
2. With another vehicle
3. With Animal
4. With obstacle

403. If your answer to question number 401 is **"Yes",** what was the consequence of the accident?

1. Death ____people
2. Death ____ animal/s
3. Serious injury _____ people
4. Minor injury _____ people
5. Property damage only

404. If your answer to question number 401 is **"Yes",** who was injured?

1. Passenger 3. Driver
2. Pedestrian

405. If your answer to question number 401 is **"Yes",** who was dead?

3. Passenger 3. Other driver
4. Pedestrian

406. If your answer to question number 401 is **"Yes",** where was the accident happen?

1. Near school
2. Around church/mosque
3. At commercial center
4. Other, specify ______________

407. If your answer to question number 401 is **"Yes",** what was the road trip?

1. Asphalt
2. None asphalt

408. If your answer to question number 401 is **"Yes",** what was the junction of the road?

1. Straight
2. Square
3. Two junctions
4. Three junctions
5. Other, specify ______________

409. If your answer to question number 401 is **"Yes",** what was the weather condition during the accident?

1. Normal weather condition
2. Rainy
3. Cloudy
4. Fog
5. Windy

410. If your answer to question number 401 is **"Yes",** at what time was the accident? Specify,___________.

Thank you very much!

Local language (*Tigrigna*) questionnaire

ቃለ መሕትት

ክፍሊ ሓደ: ማሕበራዊ፣ ኢኮኖሚያዊን ስነ-ህዝባውን ኩነታት ሹፌር

101. ዕድመኻ ክንደይ እዩ? _______ዓመት ሃይማኖትኻ እንታይ እዩ?

1. ኦርቶዶክስ
2. ካቶሊክ
3. ሙስሊም
4. ፕሮቴስታንት
5. ካሊእ ______________

102. ኩነታት ሓዳርኻ ኣብ አየናይ ይምደብ?

1. ሓዳር የብለይን
2. ብዓል ሓዳር እየ

3. ዝፈታሕኩእየ

103. ክንደይ ቆልዑ አለካ?

1. 0 ቆልዑ
2. 1-2 ቆልዑ
3. ልዕሊ 2 ቆልዑ

104. ክንደይ ናይ ስድራ ኣባላት ትሕግዝ?

1. 0 ኣባላት ስድራ
2. ካብ 1-2 ኣባላት ስድራ
3. ካብ 3-4 ኣባላት ስድራ
4. 5ን ልዕሊኡን ኣባላት ስድራ

105. ደረጃ ትምህርትኻ ኣብ አየናይ ትምደብ?

1. ምፅሓፍን ምንባብን ይክእል
2. ቀዳማይ ብርኪ
3. ካልኣይ ብርኪ
4. ዲፕሎማን ልዕሊኡን

106. ወርሓዊ መሃያኻ ክንደይ እዩ? ________ብር

107. ናይ ባዕልኻ መንበሪ ገዛ ኣለካ ዶ?

1. እወ
2. የብለይን

ክፍሊ ክልተ: ባህርያት ገናሒ መኪና/ባጃጅ

201. ናይ መጀመርታ ሓገዝ ሕክምና ስልጠና ሰልጢንካ ዶ?

1. እወ
2. ኣይፋሉን

202. ናይ ህይወት ዉሕስና አለካ ዶ?

1. እወ
2. የብለይን

203. ናይ ወንበርኻ ሓደጋ መከላኸሊ መዕጠቂ ትጥቀመሉ ዲኻ?

1. እወ

2. ኣይፋሉን

204.ን ቁፅሪ 203 መልስኻ እወ እንተኮይኑ ናይ ወንበርካ ሓደጋ መከላኸሊ መፅጠቒ ብከመይት ጥቀመሉ?

1. ኩሉግዘ
2. መብዛሕትኡ ግዘ
3. ሓሓሊፉ

205. ንቁፅሪ 203 መልስኻ ኣይፋሉን እንተኮይኑ ምኽንያትኻ እንታይ እዩ?

1. የሰልችው
2. ተበላሽዩኒ
3. ረብሓ የብሉን ኢለ ይሓስብ
4. የብለይን
5. ካሊእ ይገለፅ______________

206. ኣልኮላዊ መስተ ትሰቲዲኻ?

1. እወ
2. ኣይሰትን

207.ንቁፅሪ 206 መልስኻ እወ እንተ ኮይኑ ኣልኮላዊ መስተ ምስ ሰተኻ ኣብ ሰለስተ ስዓት ውሽጢ ናይ ምዝዋር ልምድኻ እንታይ ይመስል?

1. መብዛሕትኡ ግዘ
2. ሓሓሊፉ
3. ብጣዕሚሓ ሓሊፉ
4. ምንም ኣይገብሮን

208. ናይ ትራፊክ መምርሒ ጥሒስካ ተቐፂዕኻ 'ዶ ትፈልጥ?

1. እወ
2. ኣይፋሉን

209.ንቁፅሪ 208 መልስካ እወ እንተኮይኑ ክንደይ ግዘ ተቐፂዕካ?

1. ካብ 1-2 ግዘ
2. ካብ 3-4 ግዘ
3. ልዕሊ 4

210. ጫት ትቕሕምዲ ኻ?

1. እወ

2. ኣይፋሉን

211. ንቐፅሪ 210 መልስኻ እወ እንተኮይኑ ናይ ምቕሓም ልምድኻ እንታይ ይመስል?

1. ኩሉ ግዘ
2. መብዛሕትኡ ግዘ
3. ሓሓሊፉ

212. ንቐፅሪ 210 መልስኻ እወ እንተኮይኑ፣ ጫት እትቕሕሞ ዘለኻን ምንታይ ረብሓ እዩ?

1. ስለዘነቓቕሓኒ/ሓይሊ ስለ ዝህበኒ
2. ታሕጓስ ስለ ዝስመዐኒ
3. ነፃነት ይስመዓኒ

213.ካሊእ ይገለፅ_______ ካብ ዝትወሰነ መጠን ናህሪ ሓሊፍካ ‘ዶ ትፈልጥ?

1. እወ
2. ኣይፋሉን

214. ንቐፅሪ 213 መልስካ እወ እንተኮይኑ፣ ካብ ዝተወሰነ ናይ ናህሪ መጠን ናይ ምዝዋር ልምድኻ እንታይ ይመስል?

1. መብዛሕትኡ ግዘ
2. ሓሓሊፉ
3. ብጣዕሚ ሓሓሊፉ

215. ንቐፅሪ 213 መልስካ እወ እንተኮይኑ ፣ ካብ ዝተወሰነ ናይ ናህሪ መጠን ምዝዋር ምኽንያቱ እንታይ እዩ ትብል?

1. ተራ አቐዲመ ንምሓዝ
2. ብዙሕ ኣታዊ ንምርካብ
3. ምስ ካሊእ ታክሲ ህልኽ ስለ ዝኣትው
4. ካሊእ ይገለፅ_____________

216. እናዘወርካ እናሃለካ ስልኪ እንተተደዊልካ ከመይ ጌርካ ትቕበሎ?

1. ናህረይ ቀኒሰ ይቕበል
2. ጠጠው ኢለ ይቕበል
3. ብዘለኽዎ ናህሪ ይቕበል
4. ስልኩ ይዓፅዎ

217. ምሸት ክሳብ ክንደይ ስዓት ትሰርሕ? ክሳብ ___________ስዓት ናይ ቴሌቪዥን ወይም ኤፍኤም ፕሮግራማት ትከታተል 'ዶ?

1. እወ
2. ኣይከታተልን

218. ደረጃ መዘወሪ ፍቃድካ ክንደይ እዩ? ______ይ ደረጃ

219. ታክሲ ካብ እትገንሕ ክንደይ ዓመት ጌርካ? ________ ዓመት

220. ስልጠና መዘወሪ ፍቓድ ኣበይ ሰልጢንካ?

____

ክፍሊ ሰለስተ፡-ብዛዕባ ተሽከርካሪ ዝምልከት

301. ግልጋሎት ዘመን ናይ መኪናኻ/ባጃጅኻ ክንደይ ዓመት እዩ?_______ዓመት

302. ኣብዚ ሐዚ እዋን ናይ ኣየናይ ዓይነት ተሽከርካሪ ገናሒ ኢኻ?

1. ናይ ባጃጅ
2. ናይ ሚኒባስ

303. ናይዛ ተሽከርካሪ ብዓል ዋና መን እዩ? 1. ናተይ እያ 2. ናይ ቖፃሪየይ እያ

304. መኪናካ/ባጃጅካ ፀገም ይገጥማ ዶ? 1. እወ 2. ኣይፋሉን

305. ንቑፅሪ 304 መልስካ እወ እንተኮይኑ፣ መኪናካ/ባጃጅካ መብዛሕቲኡ ግዘ እንታይ ዓይነት ፀገም የጋጥማ?

1. ናይ ፍሬን
2. ናይ መሪ
3. ናይ መብራህቲ
4. ናይ ጎማ

ክፍሊ ኣርባዕተ፡-ብዛዕባ ኩነታት ሓደጋ

401. ኣብ ዝሓለፈ 3 ዓመት ዉሽጢ ሓደጋ ገጢሙካ ይፈልጥ ዶ?

1. እወ
2. ኣይፈልጥን

402. ንቑፅሪ 401 ሕቶ መልስካ እወ እንተኮይኑ መንቀሊ ናይ'ቲ ሓደጋ እንታይ እዩ? ካብ ሓደ መልሲ ንላዕሊ ምምራፅ ይከኣል።

1. ካብ መጠን ዝሓለፈ ናህሪ
2. ኣልኮል መስተ

3. ጫት

4. ናይ ኣጋር ዕሽሽ ምባል

5. ናይ የማን መስመር ዘይምሓዘይ

6. ንኣጋር ቅድሚያ ዘይምሃበይ

7. ስልኪ እናዛረብኩ

8. ምስ ካሊእ ተሽከርካሪ ብጣዕሚ ተፀጊዐ ብምጉዓዘይ

9. ናይ መንገዲ ፀገም

10. ናይ ተሽከርካሪ ሜካኒካል ፀገም

11. ካሊእ፣ ይገለፅ_____________

403. ንቑፅሪ 401 ሕቶ መልስኻ እወ እንተኮይኑ እቲ ግጭት ምስ መን ነይሩ?

1. ምስ ሰብ/ኣጋር

2. ምስ ካሊእ ተሽከርካሪ

3. ምስ እንስሳ

4. ምስ ግኡዝ ኣካል

404. ንቑፅሪ 401 ሕቶ መልስ እወ እንተኮይኑ ሳዕቤን ናይቲ ሓደጋ እንታይ ነይሩ?

1. ሞት በዝሒ _____ሰባት

2. ሞት በዝሒ______እንስሳት

3. ከቢድ መጉዳእቲ በዝሒ_____ሰባት

4. ቀሊል መጉዳእቲ በዝሒ_____ሰባት

5. ዕንወት ንብረት ጥራይ

405. ንቑፅሪ 401 ሕቶ መልስኻ እወ እንተኮይኑ ዝተጎድአ መን ነይሩ?

1. ተሳፋራይ

2. ኣጋር

3. ኣሽከርካሪ

406. ንቑፅሪ 401 ሕቶ መልስኻ እወ እንተኮይኑ ዝሞተ መን ነይሩ?

1. ተሳፋራይ 2. ኣጋር 3.ካሊእ ኣሽከርካሪ

407. ንቑፅሪ 401 ሕቶ መልስኻ እወ እንተኮይኑ እቲ ሓደጋ ኣበይ ከባቢ ነይሩ?

1. ኣብ ከባቢ ቤት ትምህርቲ

2. ኣብ ከባቢ ቤ/ክርስትያን ወይም መሰጊድ

3. ኣብ ከባቢ ዕዳጋ

4. ኣብ ካሊእ ቦታ

408. ንቑፅሪ 401 ሕቶ መልስኻ እወ እንተኮይኑ እቲ መንገዲ እንታይ ዓይነት ነይሩ?

1. አስፋልት መንገዲ

2. ፀፀር/ኮረኮንቺ መንገዲ

409. ንቑፅሪ 401 ሕቶ መልስኻ እወ እንተኮይኑ ናይቲ መንገዲ አከፋፍላ እንታይ ዓይነት ነይሩ ቀጥ ዝበለ

1. አደባባይ
2. ብዓል ክልተ መካፈሊ
3. ብዓል ሰለስተ መካፈሊ
4. ካሊእ፣ ይገለፅ______________

410. ንቑፅሪ 401 ሕቶ መልስኻ እወ እንተኮይኑ እንታይ ዓይነት አየር ፀባይ ነይሩ?

1. ተለመደ/ፀሓይ አየር ፀባይ
2. ዝናብ አየር ፀባይ
3. ደመናማ
4. ዕምብሮአየርፀባይ
5. ንፋሳማ አየር ፀባይ

411. ንቑፅሪ 401 ሕቶ መልስኻ እወ እንተኮይኑ እቲ ሓደጋ ኣብ ክንደይ ስዓት ነይሩ? ይገለፅ______________

የቐንየለይ!
